# Von Generation zu Generation: Mechanismen der Risikoübertragung elterlicher psychischer Erkrankungen im frühen Kindesalter

**DOI:** 10.1007/s00103-024-03978-3

**Published:** 2024-11-25

**Authors:** Anna-Lena Zietlow, Lea Krumpholtz

**Affiliations:** Professur für Klinische Kinder- und Jugendpsychologie, Fakultät für Psychologie, Institut für Klinische Psychologie und Psychotherapie, Technische Universität Dresden, Chemnitzer Str. 46, 01187 Dresden, Deutschland

**Keywords:** Elterliche Psychopathologie, Sozio-emotionale und kognitive Entwicklung, Intergenerationale Transmission, Kinder psychisch kranker Eltern, Entwicklungsrisiken, Parental psychopathology, Socio-emotional and cognitive development, Intergenerational transmission, Children of mentally ill parents, Developmental risks

## Abstract

Kinder, die mit einem psychisch kranken Elternteil aufwachsen, sind vielfältigen und weitreichenden Entwicklungsrisiken ausgesetzt. Die intergenerationale Übertragung elterlicher psychischer Störungen wird durch eine Vielzahl von Risiko- und Schutzfaktoren sowie vermittelnden Mechanismen beeinflusst, die sowohl aufseiten der Eltern und Kinder als auch im sozialen Umfeld liegen. Der Einfluss der elterlichen Psychopathologie ist in den ersten Lebensjahren besonders stark, beeinflusst aber auch die Entwicklung im Kindes- und Jugendalter und kann sich lebenslang negativ auf die psychische Gesundheit auswirken. Aufgrund der vielfältigen und langfristigen Auswirkungen auf die kindliche Entwicklung ist neben der Identifikation von Transmissionsfaktoren auch die Ableitung möglichst frühzeitiger Präventions- und Interventionsstrategien von hoher Relevanz, um die weitreichenden negativen Folgen für die Entwicklung der Heranwachsenden reduzieren zu können. Mögliche Ansatzpunkte hierfür bieten zum einen identifizierte Risiko- und Schutzfaktoren sowie vermittelnde Mechanismen zwischen elterlicher Psychopathologie und kindlicher Entwicklung. Diese Faktoren und ihre Auswirkungen auf die frühkindliche Entwicklung werden im vorliegenden narrativen Review auf der Basis des aktuellen Forschungsstandes dargestellt. Darüber hinaus werden Forschungslücken identifiziert und Implikationen für die Ableitung früher Interventionen diskutiert.

## Abstract (en)

Growing up with a mentally ill parent is associated with multiple and far-reaching developmental risks for children. The intergenerational transmission of parental mental disorders is influenced by a variety of risk and protective factors as well as mediating mechanisms both on the part of the parents and children and in the social environment. The influence of parental psychopathology is particularly strong in the first years of life, but also affects development in childhood and adolescence and can have a lifelong negative impact on mental health. Due to the diverse and long-term effects on child development, the identification of transmission factors and the development of prevention and intervention strategies as early as possible are highly relevant in order to reduce the far-reaching negative consequences for the development of the offspring. Possible starting points for this include identified risk and protective factors as well as mediating mechanisms between parental psychopathology and child development. These factors and their effects on early child development are presented in this narrative review based on the current state of research. In addition, research gaps are identified and implications for the development of early interventions are discussed.

## Hintergrund

Aktuellen Zahlen zufolge ist rund ein Viertel der Kinder zwischen 0 und 16 Jahren mit einer psychischen Erkrankung der Eltern konfrontiert [1]. Für den deutschsprachigen Raum fehlen derzeit repräsentative Daten. Schätzungen gehen jedoch davon aus, dass etwa 3–4 Mio. Kinder und Jugendliche in Deutschland bis zu ihrem 18. Lebensjahr mit einem psychisch kranken Elternteil aufwachsen [2].

Betroffene Kinder zeigen ein erhöhtes Risiko für Entwicklungs- und Verhaltensauffälligkeiten [3, 4], weisen Einschränkungen in ihrer Lebensqualität auf [5] und haben zudem ein 1,5- bis 8,4-fach erhöhtes Risiko, im Laufe ihres Lebens selbst eine psychische Störung zu entwickeln [6–8]. Aktuellen Daten zufolge liegt das allgemeine psychische Erkrankungsrisiko dieser Kinder zwischen 17 % und 55 %, wobei insbesondere elterliche affektive und Angststörungen einen erheblichen Risikofaktor darstellen [7]. Neben diesem allgemeinen Erkrankungsrisiko besteht bei diesen Kindern auch ein erhöhtes Risiko für die Entwicklung der spezifischen elterlichen psychischen Störung (konkordante Transmission). Diese konkordante Transmission zeigt sich insbesondere bei Aufmerksamkeitsdefizit‑/Hyperaktivitätsstörungen (ADHS) oder Angststörungen der Eltern (32 % bzw. 31 %), gefolgt von depressiven Störungen (14 %) und Substanzgebrauchsstörungen (9 %; [7]).

Dabei spielen vor allem die ersten Lebensjahre der Kinder eine entscheidende Rolle: Je früher das Kind mit elterlicher Psychopathologie konfrontiert wird, desto stärker sind die Auswirkungen auf die kindliche Entwicklung [3, 4, 9]. Zudem bestehen die damit einhergehenden Entwicklungsrisiken über die gesamte Lebensspanne hinweg [10]. Daher ist die Identifikation von Transmissionsfaktoren elterlicher Psychopathologie insbesondere in der frühen Kindheit grundlegend, um sowohl Präventions- als auch Interventionsstrategien ableiten zu können.

In diesem narrativen Review wird der aktuelle Forschungsstand zu Mechanismen der transgenerationalen Risikoübertragung, d. h. Faktoren, welche die erhöhte Vulnerabilität für die Entwicklung psychischer Auffälligkeiten bei Kindern psychisch kranker Eltern erklären, systematisch vorgestellt. Zur Evidenzsynthese wurde die Methode des narrativen Reviews gewählt, da diese es ermöglicht, Literatur zu einem breiten und heterogenen Themenfeld zusammenzutragen. Als Grundlage dienen vor allem systematische Überblicksarbeiten, die nach festgelegten Suchstrategien die aktuelle Evidenzlage zu einzelnen Transmissionsmechanismen oder Entwicklungsbereichen darstellen.

Neben der Darstellung der in der Literatur etablierten Modelle zur Risikoübertragung elterlicher psychischer Störungen auf die nachfolgende Generation wird im Folgenden der aktuelle Forschungsstand zu relevanten Mechanismen, Risiko- und Schutzfaktoren sowie den Folgen für die kindliche Entwicklung dargestellt. Diese Synthese wird abschließend kritisch diskutiert und zur Ableitung von Empfehlungen für Forschung und Praxis genutzt.

## Intergenerationale Transmission psychischer Störungen: Theoretische Modelle

Kinder von psychisch kranken Eltern haben ein erhöhtes Risiko, im Laufe ihres Lebens selbst psychische Auffälligkeiten zu entwickeln; jedoch zeigt sich, dass die Entwicklungsverläufe oftmals heterogen sind. Dies erklärt sich durch das komplexe Zusammenspiel von biologischen, psychischen und sozialen Faktoren sowie deren Wechselwirkungen. In der Forschung werden verschiedene theoretische Modelle diskutiert, welche die zugrunde liegenden Mechanismen und Wechselwirkungen zwischen diesen Faktoren beschreiben. Dabei ist auch die Beachtung von Risiko- und Schutzfaktoren zentral, um besser zu verstehen, wie diese Mechanismen zusammenwirken und welche Faktoren zur Resilienz beitragen können.

Das *Integrative Modell für die Transmission von Depression* von Goodman und Gotlib [11] beschreibt 4 Mechanismen, über die das Risiko übertragen werden könnte: (a) Vererbbarkeit von Depressionen, (b) angeborene dysfunktionale neuroregulatorische Mechanismen, (c) Exposition gegenüber negativen mütterlichen Kognitionen, Verhaltensweisen und Affekten sowie (d) der stressige Kontext im Leben der Kinder. Des Weiteren benennen die AutorInnen 3 Faktoren, welche das Risiko potenziell moderieren können, nämlich (a) die (psychische) Gesundheit des Vaters und sein Engagement für das Kind, (b) der Verlauf und der Zeitpunkt der Depression der Mutter sowie (c) die spezifischen Merkmale des Kindes.

**Abb. 1:**
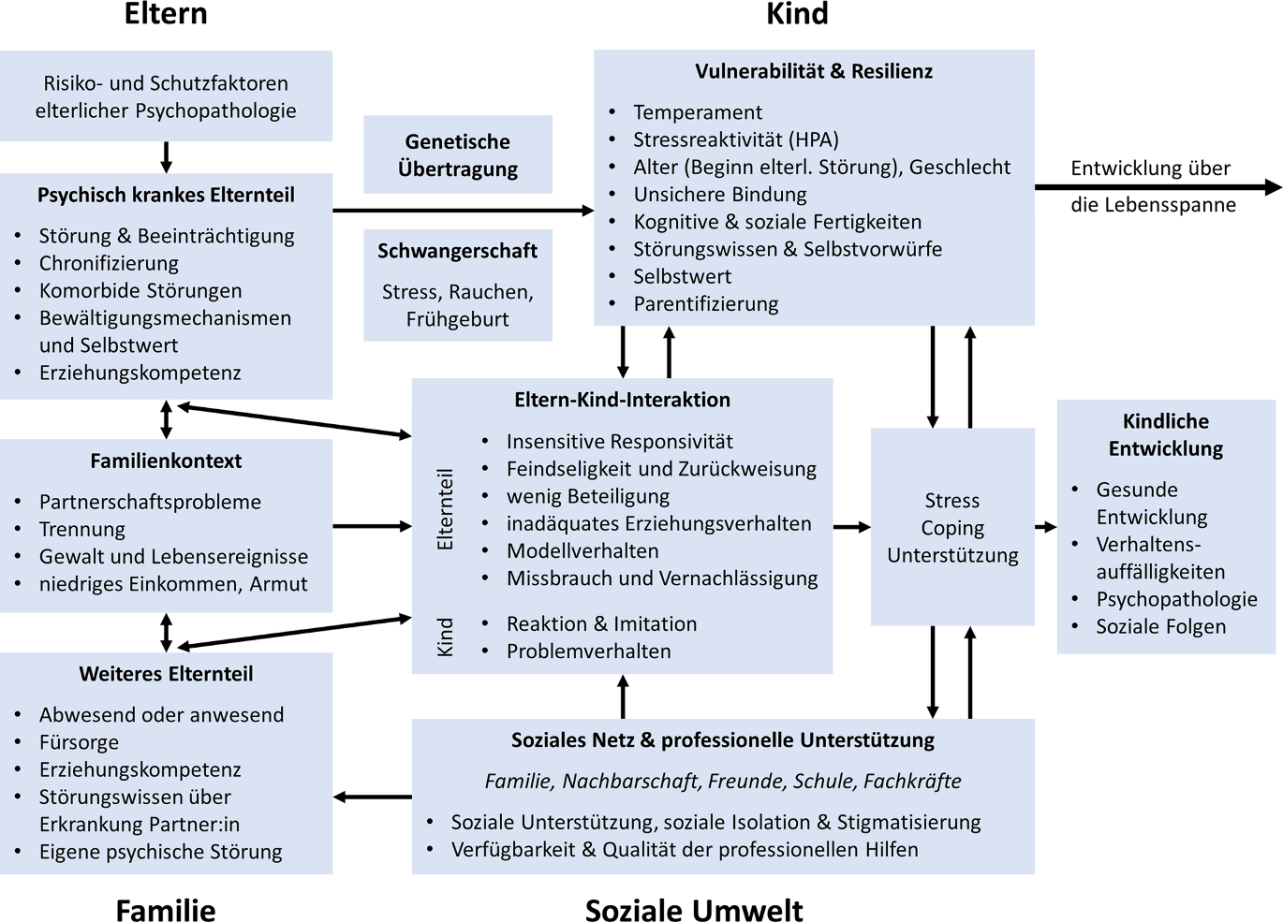
Modell der intergenerationalen Transmission psychischer Störungen. *HPA* Hypothalamus-Hypophysen-Nebennierenrinden-Achse. Übersetzt aus [12], © Taylor & Francis Ltd, http://www.tandfonline.com, 2014. Abdruck mit freundlicher Genehmigung

Das Modell erweiternd beschreiben Hosman et al. [12] die transgenerationale Weitergabe psychischer Störungen durch die Interaktion von 4 Ebenen: elterliche, familiäre, kindliche und soziale Ebene (Abb. 1). Zudem werden 5 Transmissionsmechanismen unterschieden: genetische, pränatale, familiäre und soziale Einflüsse sowie die Eltern-Kind-Interaktion. Das Modell berücksichtigt darüber hinaus, dass jede kindliche Entwicklungsphase spezifische Prozesse und Aufgaben mit sich bringt, die mit den 4 Ebenen und 5 Mechanismen interagieren. Es integriert auch Konzepte wie Äquifinalität (verschiedene Ursachen führen zu derselben Störung) und Multifinalität (ein Risikofaktor kann unterschiedliche Störungen verursachen) sowie Konkordanz (Eltern und Kind entwickeln dieselbe Störung; [8]).

## Transmissionsmechanismen

Das Modell von Hosman et al. [12] führt heterogene kindliche Entwicklungsverläufe auf im vorangegangenen Abschnitt beschriebene Transmissionsmechanismen zurück, die zwischen Risikofaktoren und Entwicklungsausgängen vermitteln. Zu betonen ist, dass eine eindeutige Abgrenzung voneinander häufig nicht möglich ist, da die Faktoren in wechselseitiger Beziehung zueinanderstehen.

### Genetische und epigenetische Faktoren

Kinder psychisch kranker Eltern haben sowohl ein erhöhtes allgemeines (unspezifisches) psychisches Erkrankungsrisiko als auch eine erhöhte Vulnerabilität, die spezifische elterliche Erkrankung zu entwickeln (konkordante Transmission; [6, 7]). Dies verdeutlicht unter anderem die genetische Komponente psychischer Störungen. Forschungsergebnisse deuten darauf hin, dass der Zusammenhang zwischen pränatalen depressiven Symptomen der Mutter und psychischen Störungen des Kindes zum Teil auf genetische Faktoren zurückzuführen ist, die intergenerational weitergegeben werden [13]. Es ist wichtig zu betonen, dass lediglich eine erhöhte Vulnerabilität für die Entwicklung einer Störung vererbt wird, die dann in Wechselwirkung mit Umweltfaktoren die Entwicklung einer psychischen Störung begünstigt [14]. Die Heritabilitätsrate, d. h. der Anteil der Varianz, der durch genetische Faktoren erklärt werden kann, ist je nach psychischer Störung unterschiedlich. Während genetische Einflüsse bei schizophrenen Störungen (81 %), ADHS (75 %) und bipolaren Störungen (75 %) stark ausgeprägt sind, werden Suchterkrankungen (57–67 %), Depressionen (37 %) und Angststörungen (30–50 %) mit einem moderaten Einfluss genetischer Faktoren in Verbindung gebracht [15, 16].

### Peripartale Faktoren: Intrauterine und neonatale Einflüsse

Psychische Erkrankungen in der Schwangerschaft gehen mit einem höheren Risiko für Mutter und Kind einher [17]. Dabei tragen frühe neuronale und psychophysiologische Veränderungen zu einer erhöhten Vulnerabilität der Kinder für die Entwicklung psychischer Störungen bei [14, 18]. So wurden beispielsweise peripartale psychische Störungen mit einer erhöhten kindlichen Stresssensitivität, Veränderungen in Stressregulationssystemen sowie in der Struktur und Konnektivität relevanter Hirnareale bei Säuglingen in Verbindung gebracht [17, 18]. Ein höheres Risiko für Mutter und Kind geht auch damit einher, dass Schwangere mit psychischen Erkrankungen seltener an Vorsorgeuntersuchungen teilnehmen und ein erhöhtes Risiko für den Konsum psychotroper und damit potenziell schädigender Substanzen auch für das Kind haben [19]. Psychische Störungen bei Schwangeren erhöhen zudem das Risiko für Geburtskomplikationen wie Frühgeburt, niedriges Geburtsgewicht oder eine für das Gestationsalter zu geringe Körpergröße [17, 19]. Erhöhter Stress in der Schwangerschaft, u. a. ausgelöst durch psychische Störungen, ist mit negativen Auswirkungen auf die kognitive und emotionale Entwicklung [20] und die physiologische Regulationsfähigkeit des Kindes [21] sowie mit einer beeinträchtigten Eltern-Kind-Interaktion assoziiert [22]. Darüber hinaus erhöhen präpartale psychische Störungen das Risiko für die Entwicklung einer posttraumatischen Belastungsstörung durch das Geburtserlebnis [23], die wiederum ein Risiko für die kindliche Entwicklung darstellen kann [24].

### Eltern-Kind-Interaktion

In der Forschung wird die Eltern-Kind-Interaktion als ein zentraler Mechanismus bei der Transmission psychischer Störungen diskutiert [25, 26]. Sie spielt u. a. eine bedeutende Rolle für die Entwicklung der kindlichen Regulationsfähigkeiten [27] und auch der Bindungssicherheit [28]. Das Interaktionsverhalten von Eltern mit psychischen Störungen ist häufig durch eine geringere elterliche Feinfühligkeit gekennzeichnet, was sich in einem wenig responsiven und passiven Interaktionsstil oder intrusivem bis feindseligem Verhalten zeigen kann [21, 29].

Auch das elterliche Bonding, d. h. die erste emotionale Bindung eines Elternteils an das (ungeborene) Kind, ist bei psychisch kranken Eltern, insbesondere bei depressiven Symptomen und Angststörungen, eingeschränkt und mit langfristigen negativen Folgen für die sozioemotionale, verhaltens- und temperamentbezogene Entwicklung verbunden [30, 31]. Gleichzeitig können ein gutes mütterliches Bonding und kindliche Bindungssicherheit die negativen Auswirkungen postpartaler Depressionen auf die Mutter-Kind-Interaktion abpuffern [32].

Erziehungsfertigkeiten stehen im Zusammenhang mit elterlichen Emotionsregulationskompetenzen [33, 34], die im Kontext psychischer Störungen beeinträchtigt sein können [35]. Dabei gehen höhere Emotionsregulationskompetenzen mit einem positiveren Erziehungsverhalten einher [34]. Umgekehrt sind psychische Störungen mit einem erhöhten Risiko für negatives Erziehungsverhalten wie erhöhte Feindseligkeit, Ablehnung, Vernachlässigung und höhere Verhaltenskontrolle verbunden [36]. Diese negativen Erziehungspraktiken sind mit Ängsten, Depressionen, internalisierenden Problemen und emotionaler Dysregulation aufseiten der Kinder assoziiert [37]. Schließlich stellen diese psychischen Folgen aufseiten der Kinder im Sinne eines Teufelskreises zusätzliche Herausforderungen für die ohnehin schon häufig verminderte Erziehungskompetenz psychisch kranker Eltern dar. Dagegen kann ein positives Erziehungsverhalten des gesunden Elternteils die entwicklungsrelevanten Risiken der psychischen Störung des erkrankten Elternteils reduzieren [14].

Die im Kontext psychischer Störungen reduzierten elterlichen Erziehungsfertigkeiten können mit einem erhöhten Risiko für Kindesmisshandlung einhergehen, allerdings ist die Studienlage hierzu nicht eindeutig [38, 39]. Eine dysfunktionale Emotionsregulation als transdiagnostischer Faktor elterlicher Psychopathologie hingegen wird mit einem erhöhten Risiko für Kindesmisshandlung in Verbindung gebracht [40]. Auch Misshandlungserfahrungen der Eltern in der eigenen Kindheit stellen einen Risikofaktor für Kindesmisshandlung dar [41]. Frühe aversive Erfahrungen erweisen sich als robuster Risikofaktor für die Entwicklung psychischer Störungen über die Lebensspanne und sind nach Kontrolle genetischer und umweltbedingter Einflüsse für bis zu 41 % der häufigsten psychischen Erkrankungen verantwortlich [42, 43].

### Familiäre Faktoren

Elterliche Modelle unangemessener Emotionsregulation oder Stressbewältigung können in Familien mit psychisch krankem Elternteil u. a. durch den Prozess der Beobachtung (sog. Modelllernen) weitergegeben werden. Für die Entwicklung funktionaler Emotionsregulationsstrategien sind Kinder auf die Unterstützung ihrer primären Bezugspersonen angewiesen. Nach der sozialkognitiven Lerntheorie fungieren Eltern als Rollenmodelle im Umgang mit eigenen Emotionen und schaffen gleichzeitig Lernerfahrungen darüber, wie sie beispielweise auf negative Emotionen seitens des Kindes reagieren [44]. Psychische Störungen gehen jedoch in vielen Fällen mit Einschränkungen in der Emotionsregulation [35] und damit mit einem weniger unterstützenden elterlichen Verhalten bei der Sozialisation von Emotionen einher [44].

Darüber hinaus sind Eltern Rollenmodelle in Bezug auf Konflikt- und Problemlöseverhalten [44]. Die dafür bedeutsame Emotionsregulationsfähigkeit der Eltern geht nicht nur über den direkten Kontakt mit dem Kind, sondern auch über die Paarinteraktion in das Lernrepertoire der Kinder ein. In Familien mit einem psychisch kranken Elternteil sind destruktive Paarkonflikte und auch Paargewalt häufiger [45] und ein wichtiger Prädiktor für Anpassungsschwierigkeiten des Kindes [46, 47]. Dabei hat nicht die Häufigkeit von Paarkonflikten per se Auswirkungen auf die kindliche Entwicklung, sondern der destruktive Umgang mit Letzteren [46]. Kinder zeigen zudem stärkere psychische Verhaltensauffälligkeiten, wenn die Familienfunktionalität nicht nur aufgrund dysfunktionaler Konflikte eingeschränkt ist, sondern bspw. auch der Alltag von fehlender Struktur geprägt ist [48].

Ein weiterer für die Entwicklung hochrelevanter Faktor ist die Rollenumkehr. Dabei übernehmen Kinder Aufgaben und Pflichten, die eigentlich von ihren Eltern erfüllt werden sollten, oder leisten unangemessenen emotionalen Beistand. Diese „Parentifizierung“ wird in der Literatur mit einer erhöhten psychischen Belastung und der Entwicklung kindlicher psychischer Störungen in Verbindung gebracht [49].

Eltern mit psychischen Erkrankungen erleben erhöhten Stress durch die Elternschaft verglichen mit Eltern ohne psychische Symptomatik [21, 50]. Elterliches Stresserleben steht mit kindlichen psychischen Problemen in Zusammenhang [26]. Zum einen können dysfunktionale Strategien der Stressbewältigung von den Kindern im Sinne des Modelllernens übernommen werden. Andererseits zeigen gestresste Eltern häufiger dysfunktionales Erziehungsverhalten, möglicherweise unter anderem aufgrund der durch Stress zusätzlich beeinträchtigten Fähigkeit zur funktionalen Emotionsregulation [33].

### Soziale Faktoren

Auf Ebene des sozialen Umfeldes erweisen sich Entstigmatisierung und Aufklärung über psychische Störungen als bedeutsam. Die Vermittlung eines entwicklungsgerechten Wissens über die elterliche Erkrankung kann Schuld- und Schamgefühle der Kinder und insgesamt Entwicklungsrisiken vermindern [51]. Mangelnde soziale Unterstützung sowie Einsamkeit und soziale Isolation gefährden die psychische Gesundheit von Kindern psychisch kranker Eltern. In diesem Zusammenhang spielen stabile außerfamiliäre Bezugspersonen eine wichtige Rolle [43].

## Risiko- und Schutzfaktoren der Transmission elterlicher psychischer Erkrankungen

Zu den elterlichen Risikofaktoren gehören ein ungünstiger Krankheitsverlauf wie Chronifizierung der Störung, Schweregrad der Symptomatik und das Vorliegen von Komorbiditäten [52]. Darüber hinaus stellen eine subjektiv als sehr hoch empfundene elterliche Krankheitsbelastung sowie eine dysfunktionale Krankheitsverarbeitung Risikofaktoren für die kindliche Entwicklung dar [53].

Zu den kindlichen Risikofaktoren zählen das Alter und Geschlecht des Kindes. Je jünger die Kinder bei (Erst‑)Manifestation der elterlichen psychischen Störung sind, desto höher ist das Risiko für die Entwicklung von Verhaltensauffälligkeiten oder einer psychischen Störung [3, 9]. Die Befunde zu einem geschlechtsspezifischen Risiko sind bislang uneinheitlich. Studien zu depressiven Störungen bei Müttern deuten darauf hin, dass Mädchen anfälliger für die Entwicklung internalisierender Probleme sind und dass es entweder keine geschlechtsspezifischen Unterschiede oder mehr externalisierende Probleme bei Jungen gibt [3, 8, 9, 54]. Auch das kindliche Temperament, insbesondere negative Emotionen und eine beeinträchtigte Selbstregulation in der frühen Kindheit, stellt einen Risikofaktor für die Entwicklung psychischer Störungen im Kindes- und Jugendalter dar [55].

Im familiären Kontext hat sich in der bisherigen Forschung ein niedriger sozioökonomischer Status, einhergehend mit weiteren Faktoren wie Bildung, Finanzen, Lebensstandard und Wohnqualität, insbesondere in den ersten Lebensjahren als robuster Risikofaktor für die Entwicklung psychischer Störungen erwiesen [43].

Zusammenfassend kann man festhalten, dass die beschriebenen Transmissionsmechanismen mit elterlichen wie auch kindlichen Risiko- und Schutzfaktoren interagieren. Diese Interaktionen tragen dazu bei, dass sich die Auswirkungen elterlicher psychischer Störungen auf die kindliche Entwicklung unterschiedlich manifestieren. Das genaue Verständnis dieser Zusammenhänge ist entscheidend für die Entwicklung von Interventionen, die Risiken mindern und Resilienz bei betroffenen Kindern fördern sollen.

## Entwicklungsrisiken

Das Aufwachsen mit einem psychisch kranken Elternteil hat erhebliche Folgen für die Entwicklung und psychische Gesundheit von Kindern [3, 8, 14, 17]. Am häufigsten sind Kinder mit elterlichen Depressionen (17,5 %) und Angststörungen (7,2 %) der Eltern konfrontiert, gefolgt von Substanzgebrauchsstörungen (0,26 %) und affektiven Psychosen oder einer bipolaren Symptomatik (0,24 %) [1]. Aufgrund der hohen Prävalenz wird im Folgenden vor allem auf die Auswirkungen dieser Störungen eingegangen. Nach Entwicklungsbereichen geordnet, werden die Zusammenhänge zwischen elterlichen Störungen innerhalb der einzelnen Bereiche in Abhängigkeit vom Zeitpunkt der Störung und dem Alter der Kinder dargestellt.

### Sozioemotionale Entwicklung

Elterliche Psychopathologie geht mit Einschränkungen der sozioemotionalen Entwicklung einher [3, 4, 56, 57]. Emotionale und Verhaltensauffälligkeiten von Kindern werden in der Literatur vorwiegend als internalisierend und externalisierend konzeptualisiert. Unter internalisierenden Problemen werden Symptome von Depressionen oder Ängsten zusammengefasst, während externalisierende Auffälligkeiten Verhaltensstörungen wie hyperkinetische Störungen und Störungen des Sozialverhaltens umfassen.

Peripartale Depressionen und Angststörungen begünstigen kindliche sozioemotionale Probleme [3, 10, 17, 54, 58]. Betroffene Kinder haben im Vergleich zu Kindern psychisch gesunder Mütter ein 1,5- bis 2‑fach erhöhtes Risiko, vor dem 18. Lebensjahr sozioemotionale Auffälligkeiten zu entwickeln [58]. Erste Studien deuten darauf hin, dass sich negative Auswirkungen auf die sozioemotionale Kindesentwicklung auch für väterliche peripartale Depressionen zeigen [59]. Im Vorschulalter weisen diese Kinder höhere internalisierende und externalisierende Verhaltensauffälligkeiten auf [60] und sind in Interaktionen weniger responsiv [32]. Kinder von Eltern mit Alkoholmissbrauch tragen ebenfalls ein höheres Risiko, psychische und Verhaltensstörungen zu entwickeln [61]. Dabei sind Befunde bzgl. der Auswirkungen des Schweregrades des Alkoholkonsums uneinheitlich.

Auch im weiteren Entwicklungsverlauf zeigt die Forschung, dass mütterliche Depressionen, die in Kindheit oder Jugend erlebt werden, mit erhöhten internalisierenden und externalisierenden Verhaltensauffälligkeiten, allgemeiner Psychopathologie, antisozialem Verhalten, erhöhtem negativen Affekt und vermindertem positiven Affekt einhergehen [3, 62]. Ein ähnliches Bild zeigt sich auch für die Auswirkungen elterlicher Angststörungen, die insbesondere im mittleren Kindesalter mit einem erhöhten Risiko negativer sozioemotionaler Entwicklungsverläufe verbunden sind [4]. Für den elterlichen Substanzkonsum zeigen Längsschnittstudien schwache Zusammenhänge zwischen elterlichem Substanzkonsum und kindlichem Wohlbefinden [57]. Der Zusammenhang ist bei elterlichem Drogenkonsum deutlicher als bei Alkohol- oder Tabakkonsum, unabhängig davon, wie lange das Kind dem elterlichen Konsum ausgesetzt war. Die Autoren betonen die Bedeutung sozioökonomischer Drittvariablen in diesem Zusammenhang [57].

Während externalisierende Verhaltensauffälligkeiten gleichermaßen mit psychischen Problemen von Mutter und Vater in Zusammenhang gebracht wurden, legen metaanalytische Befunde einen stärkeren Zusammenhang zwischen internalisierenden Problemen und mütterlicher psychischer Symptomatik nahe [56].

### Bindungssicherheit

Psychische Erkrankungen werden häufig mit Bindungsunsicherheiten, ängstlichem oder vermeidendem Interaktionsverhalten in Verbindung gebracht [63]. Der Bindungsstil psychisch kranker Eltern kann transgenerational weitergegeben werden, jedoch erwiesen sich die kleinen bis moderaten Zusammenhänge zuletzt niedriger als ursprünglich in der Literatur angenommen [64]. Zudem kann elterliche Sensitivität, die sich bei psychischen Erkrankungen häufig als eingeschränkt erweist, diese intergenerationale Weitergabe der Bindungssicherheit nur teilweise erklären [64]. Unsicheres kindliches Bindungsverhalten steht wiederum in Zusammenhang mit beeinträchtigten kindlichen Entwicklungsmaßen [65] und einem erhöhten Entwicklungsrisiko psychischer Störungen über die Lebensspanne hinweg [66].

### Kognitive und Sprachentwicklung

Peripartale Depressionen und Angststörungen wirken sich auch nachteilig auf die kognitive (u. a. Gedächtnis, Leistungs-IQ und verbaler IQ) und sprachliche Entwicklung im frühen Kindesalter aus [10, 17, 67]. Insbesondere postpartale Erkrankungen stehen mit der kognitiven und sprachlichen Entwicklung in mäßigem bis starkem Zusammenhang [10]. Dabei zeigt sich vor allem bei Jungen ein negativer Einfluss auf den IQ-Wert verglichen mit Mädchen [67]. Es gibt erste Hinweise darauf, dass der Zusammenhang zwischen peripartaler Depression und der kognitiven Entwicklung der Heranwachsenden zumindest teilweise auf die verminderte Responsivität von Müttern mit Depressionen im Postpartalzeitraum zurückgeführt werden könnte [67]. Der Zusammenhang zwischen mütterlicher peripartaler Depression und der kindlichen Exekutivfunktionen (bspw. Planen, Aufmerksamkeit, Inhibition) erwies sich dagegen als sehr gering [68]. Für die im Rahmen elterlicher psychischer Störungen gehäuft auftretende verzögerte Sprachentwicklung scheint insbesondere die Chronifizierung der elterlichen Symptomatik eine Rolle zu spielen [10, 52]. Kinder von Müttern mit postpartaler Depression haben zudem ein erhöhtes Risiko für verringerte schulische Leistungen bis ins Jugendalter [69].

## Synthese des Forschungsstands zur frühkindlichen Entwicklung im Kontext elterlicher psychischer Erkrankungen

Epidemiologischen Studien nach ist jedes 4. bis 5. Kind bis zum 18. Lebensjahr von einer psychischen Störung der Eltern betroffen [1]. Elterliche psychische Erkrankungen stellen einen bedeutsamen Risikofaktor für die kindliche Entwicklung dar und können sich auf alle Entwicklungsbereiche und die gesamte Lebensspanne auswirken.

Das Modell der intergenerationalen Transmission von Psychopathologie nach Hosman et al. [12] unterstreicht, dass ein komplexes Zusammenspiel genetischer, pränataler und umweltbedingter Einflüsse das Risiko für ungünstige kindliche Entwicklungsverläufe bedingt. Die Risiko- und Schutzfaktoren auf elterlicher und kindlicher Seite beeinflussen die kindlichen Entwicklungsverläufe. Dies geschieht im komplexen Zusammenspiel mit zentralen Transmissionsmechanismen, unter denen die Eltern-Kind-Interaktion eine bedeutsame Rolle einnimmt. Das elterliche Interaktionsverhalten hat einen starken Einfluss sowohl auf die Entwicklung grundlegender sozioemotionaler Kompetenzen, wie bspw. Selbstregulationsfertigkeiten und Emotionsregulation, als auch auf die Bindungssicherheit des Kindes. Daneben spielen die vorhandene soziale Unterstützung und individuelle Stress- und Copingfähigkeiten eine vermittelnde Rolle bei der Transmission elterlicher psychischer Störungen.

Die Zusammenschau des aktuellen Forschungsstandes weist auf relevante Forschungslücken hin. So steht die Forschung zu Vätern und ihrer Bedeutung für die kindliche Entwicklung noch am Anfang, Gleiches gilt für Familien in nichttraditionellen Familienformen. Bisher werden in Studien auch meist nur einzelne Familienmitglieder und nicht das gesamte Familiensystem betrachtet. Während zu elterlichen Depressionen und Angststörungen vergleichsweise viele Studien vorliegen, sind die Auswirkungen anderer Störungsbilder, wie z. B. Persönlichkeitsstörungen, die ebenfalls mit Beeinträchtigungen der elterlichen Emotionsregulations- oder Interaktionsfähigkeiten einhergehen, wenig erforscht. Bisher wurden in Studien überwiegend einzelne Transmissionsmechanismen betrachtet, das Zusammenspiel unterschiedlicher Transmissions- oder Risiko- bzw. Schutzfaktoren im Längsschnitt aber nur unzureichend untersucht. Dies erschwert es, valide Aussagen über die Relevanz und Auswirkungen einzelner Transmissionsmechanismen für die kindliche Entwicklung zu treffen. Für den deutschsprachigen Raum fehlen zudem aktuelle repräsentative Prävalenzdaten zu Kindern psychisch kranker Eltern, die zu einer Sensibilisierung für die Dringlichkeit der Aufmerksamkeit für diese Hochrisikogruppe beitragen könnten.

Die Form des narrativen Reviews ist mit einer Reihe von Limitationen verbunden. Diese Methode der Evidenzsynthese wurde gewählt, um einen möglichst breiten Überblick zu den vielfältigen Transmissionsmechanismen und Entwicklungsbereichen zu geben. Allerdings obliegt die Literaturauswahl des Reviews den Autorinnen und basiert – anders als bei systematischen Reviews – nicht auf einer zuvor festgelegten Suchstrategie. Es wurde von den Autorinnen darauf geachtet, systematische Überblicksarbeiten zu zitieren, um eine verzerrte Darstellung, wie sie bei der Auswahl von Einzelstudien entstehen kann, zu reduzieren.

## Implikationen und Ausblick

Angesichts der gravierenden und nachhaltigen Auswirkungen elterlicher psychischer Störungen auf die kindliche Entwicklung und die gesamte Lebensspanne ist eine niedrigschwellige, auf die individuellen Bedürfnisse der Familie zugeschnittene Unterstützung dringend erforderlich. Diese Unterstützung sollte langfristig angelegt sein und erfordert häufig eine multidisziplinäre Zusammenarbeit. Ein frühzeitiges Screening zur Identifizierung gefährdeter Familien, bereits während der Schwangerschaft, sowie niedrigschwellige Präventions- und Interventionsangebote nach dem Stepped-Care-Ansatz können entscheidend dazu beitragen, negative Folgen zu mindern und betroffene Familien zu stärken.

Darüber hinaus zeigt die Forschung, dass bei Einschränkungen der Eltern-Kind-Beziehung eine alleinige störungsspezifische Behandlung der mütterlichen depressiven Störung nicht mit ausreichenden positiven Effekten auf die kindliche Entwicklung, die Erziehungskompetenz oder die Mutter-Kind-Beziehung einhergeht [70, 71]. Dies unterstreicht, dass bekannte Transmissionsfaktoren wie elterliches Stresserleben und Partnerschaftsprobleme neben der Eltern-Kind-Interaktion und der kindlichen Bindungssicherheit in Interventionen adressiert werden sollten [26, 50, 72, 73]. Metaanalytische Befunde legen insbesondere eine Wirksamkeit von Interventionen nahe, die sich sowohl an Eltern als auch an das Kind richten [74]. Der Einbezug aller Familienmitglieder wird beispielsweise im Interventionsansatz CHIMPs („children of mentally ill parents“) praktiziert, der aktuell evaluiert wird [75].

Für die Forschung ergibt sich aus der vorliegenden Synthese die Dringlichkeit einer empirischen Modellprüfung des Zusammenwirkens verschiedener Transmissionsmechanismen wie auch Schutz- und Risikofaktoren [76]. Diese wird beispielsweise durch das Forschungskonsortium der COMPARE-Studie („children of mentally ill parents at risk evaluation“; [25]) im Rahmen verschiedener Teilprojekte mittels der längsschnittlichen Begleitung von Familien adressiert. Die Präventions- und Interventionsplanung setzt neben diesem wissenschaftlichen Verständnis auch eine bisher ausstehende gesundheitsökonomische Evaluation der Eltern-Kind-Versorgung in Deutschland voraus. Eine Integration von Präventions- und Interventionsangeboten für betroffene Familien in die Regelversorgung lässt sich jedoch nur über eine ausreichende empirische Fundierung begründen.
